# Transcriptomic analysis of loss of Gli1 in neural stem cells responding to demyelination in the mouse brain

**DOI:** 10.1038/s41597-021-01063-x

**Published:** 2021-10-28

**Authors:** Jayshree Samanta, Hernandez Moura Silva, Juan J. Lafaille, James L. Salzer

**Affiliations:** 1 Department of Neuroscience and Physiology, Neuroscience Institute, New York University School of Medicine, New York, NY 10016 USA; 2 The Kimmel Center for Biology and Medicine of the Skirball Institute, New York University School of Medicine, New York, New York 10016 USA; 3 Present Address: Stem Cell and Regenerative Medicine Center, Department of Comparative Biosciences, School of Veterinary Medicine, University of Wisconsin-Madison, Madison, WI 53706 USA

**Keywords:** Multiple sclerosis, Neural stem cells

## Abstract

In the adult mammalian brain, Gli1 expressing neural stem cells reside in the subventricular zone and their progeny are recruited to sites of demyelination in the white matter where they generate new oligodendrocytes, the myelin forming cells. Remarkably, genetic loss or pharmacologic inhibition of Gli1 enhances the efficacy of remyelination by these neural stem cells. To understand the molecular mechanisms involved, we performed a transcriptomic analysis of this Gli1-pool of neural stem cells. We compared murine NSCs with either intact or deficient Gli1 expression from adult mice on a control diet or on a cuprizone diet which induces widespread demyelination. These data will be a valuable resource for identifying therapeutic targets for enhancing remyelination in demyelinating diseases like multiple sclerosis.

## Abstract (summary)

**Table:** 

Measurement(s)	RNA
Technology Type(s)	RNA sequencing
Factor Type(s)	genotype • diet
Sample Characteristic - Organism	Mus musculus
Sample Characteristic - Location	United States

Machine-accessible metadata file describing the reported data: 10.6084/m9.figshare.16543485

## Background & Summary

Multiple Sclerosis (MS) is the most common cause of neurological disability in young adults^1^ and is characterized by inflammatory demyelination leading to axonal injury. In addition to preventing immune-mediated demyelination, a major therapeutic goal in MS is to enhance new myelin sheath formation by remyelination which prevents the loss of axons and restores function^2–4^. The nervous system is capable of remyelination as observed in autopsy specimens from MS patients characterized by short, thin myelin sheaths^5–8^. However, remyelination ultimately fails, particularly in MS patients with progressive disease^8,9^ and the factors that limit remyelination remain poorly understood^9,10^.

There are three sources of remyelinating cells in the adult brain - neural stem cells (NSCs)^11–13^, oligodendrocyte progenitor cells (OPCs)^14–16^ and mature oligodendrocytes^17,18^. NSCs reside in the subventricular zone (SVZ) while the OPCs and oligodendrocytes are present throughout the parenchyma in the adult brain and are recruited locally to regenerate myelin in demyelinated lesions^15,19^. Furthermore, NSCs can promote remyelination either by replenishing the adult OPCs recruited into lesions, or as a direct source of oligodendrocytes themselves. Consistently, prior studies have shown that NSCs in the SVZ are activated by local demyelination, migrate out and differentiate into OPCs that generate remyelinating oligodendrocytes^11,13,14,20^. Indirect marker studies of postmortem brains further suggest that NSCs proliferate during acute attacks of MS and are a source of remyelinating cells in the human brain^12,21,22^. More importantly, ablating NSCs results in axonal loss highlighting their significant functional contribution to remyelination^23^. Taken together, these studies suggest remyelination by NSCs protects axons. Thus, enhancing remyelination by NSCs is expected to provide a neuroprotective strategy in MS.

The adult SVZ is comprised of a functional, quiescent neural stem cell population, which are multipotent, divide slowly, self-renew, and give rise to proliferating, transit-amplifying cells^24^. There is considerable heterogeneity among these NSCs, indicated by their expression of specific transcription factors and generation of distinct cells during homeostasis and in response to injury^25^. One NSC subset, which is enriched in the ventral SVZ, expresses the transcription factor Gli1 and generates interneurons in the olfactory bulb and astrocytes in the healthy mouse brain^26,27^. This population of NSCs is also present in the human SVZ^27^ as well as in NSCs derived from human induced pluripotent stem cells and human embryonic stem cells^28^. Remarkably, and in contrast to its fate in the healthy adult brain, this Gli1 pool of NSCs is specifically recruited to the white matter in response to demyelination and goes on to differentiate into remyelinating oligodendrocytes^20,27,29^. Inhibition of Gli1 in these cells further enhances their remyelination potential and is neuroprotective resulting in functional improvement in mouse models of MS^27,28^. Importantly, Gli1 expressing stem-like cells respond to injury in other organs like the liver, lung, bone marrow, kidney and heart with loss of Gli1 resulting in attenuated injury in these organs^30–37^. Thus, inhibition of Gli1 is potentially an important therapeutic strategy for regenerative medicine and the molecular mechanisms by which Gli1 affects stem cell repair may be shared in many different types of tissues.

**Fig. 1:**
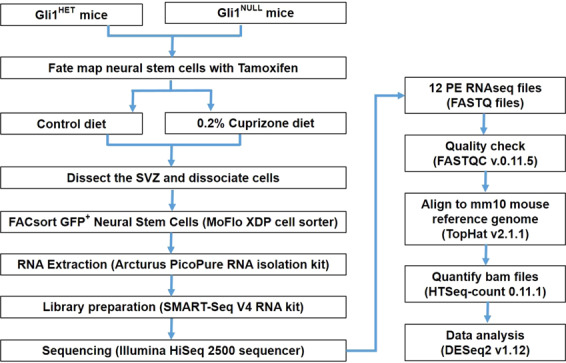
Schematic overview of the study design. The flowchart demonstrates the experimental design and data analysis.

We performed a transcriptomic analysis to identify Gli1-regulated genes that affect NSC recruitment and generation of remyelinating oligodendrocytes by comparing the mRNA expression between NSCs with genetic loss of Gli1 and those with intact Gli1 expression from both healthy and demyelinated adult mouse brains (Fig. 1). These data will be valuable not only for future remyelination studies but may also provide mechanistic insights into the shared principles of regeneration across organs.

## Methods

### Fate-mapping of neural stem cells and demyelination

All the mice had access to food and water *ad libitum* and were housed in a room with 12 hour dark/light cycle. The genetically modified mice belonged to C57bl/6 strain and were maintained according to protocols approved by New York University Medical Center’s IACUC. The *Gli1*^*CreERT2/*+^ mice (Jax # 007913) which have CreERT2 knocked into the Gli1 locus were bred with the reporter Rosa-CAG-EGFP-LoxP (RCE) (Jax# 032037) mice to generate *Gli1*^*HET*^ (*Gli1*^*CreERT2/*+^;*RCE*) with intact *Gli1* expression and *Gli1*^*NULL*^ (*Gli1*^*CreERT2/CreERT2*^;*RCE*) mice with global knockout of *Gli1*. To permanently label the Gli1 pool of NSCs with GFP, we administered 5 mg tamoxifen (Sigma) in corn oil on alternate days for a total of four intraperitoneal injections to 10 week old mice^27,38^. We did not observe any labeling in the absence of tamoxifen administration. Mice used for GFP negative controls received intraperitoneal injections of corn oil without Tamoxifen. A week after the last dose of tamoxifen/corn oil, the demyelinated group was fed 0.2% cuprizone in the chow for 3 weeks while the healthy control group remained on regular chow. Cuprizone diet induces apoptosis of mature oligodendrocytes leading to maximum demyelination of the white matter corpus callosum after 5 weeks of continuous feeding^39^. Here, we used 3 weeks of cuprizone diet for our analysis since we previously found that *Gli1*^*NULL*^ NSCs in the SVZ have significantly higher proliferation at this timepoint compared to *Gli1*^*HET*^ SVZ, suggesting that maximal activation of NSCs coincides with this early stage of demyelination^27,40^.

### Dissection of SVZ

Brain tissues were harvested from 6 mice (3 Males and 3 Females) per genotype (*Gli1*^*HET*^ and *Gli1*^*NULL*^) per diet (cuprizone and control) after euthanizing them with CO_2._ The brain was removed gently out of the skull and immediately transferred to a Petri dish chilled on ice and placed with the dorsal surface facing up on a chilled acrylic mouse brain matrix. The brains of mice were dissected according to previously published protocols^38^. Coronal sections of 1 mm thickness were made from the rostral end of the brain up to the dorsal hippocampus posteriorly, using a chilled sharp razor blade. The SVZ was identified as a thin layer of tissue lining the medial and lateral walls of the lateral ventricles and carefully dissected under a microscope from each coronal brain section.

### Dissociation and FACsorting of neural stem cells

For each RNA sample, the SVZ from one female and one male mice was pooled together. Overall, we had 3 RNA samples/genotype from control diet group and 3 RNA samples/genotype from cuprizone diet group in this study. The SVZ was then dissociated into single cell suspension following the manufacturer’s protocol in the papain dissociation kit (Worthington Biochemicals # LK003150)^41^. Briefly, the dissected SVZ tissue was minced using iris scissors and digested by incubating in a solution containing 20 Units/ml papain and 0.005% DNase for 30 minutes at 37 °C. Subsequently, the papain was inactivated with albumin-ovomucoid inhibitor and the digested tissue was needle triturated to obtain a cell suspension^41^.

**Fig. 2:**
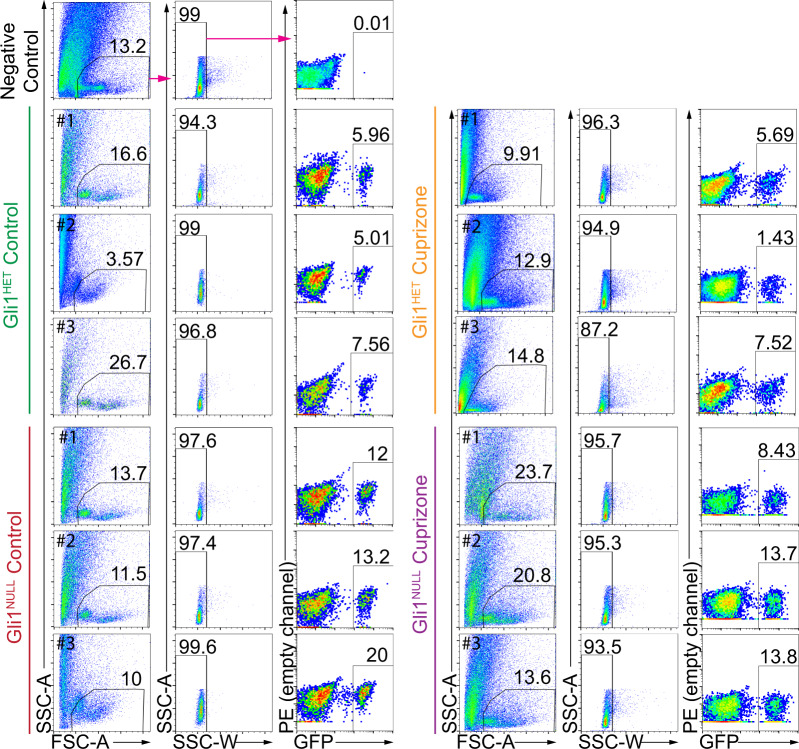
Flow cytometry analysis displaying the gating strategy used to sort fate-mapped neural stem cells. The negative control (top row) displays the gating strategy highlighted by the magenta arrows. The same strategy was applied for all samples in the figure. Fate-mapped, (i.e. GFP+) neural stem cells were purified from each of the samples depicted in the figure and processed for RNAseq. Percentage of gated cells is displayed above the selected area and the sample number is indicated in the top left corner of each dot plot. SSC- Side scatter, FSC- Forward scatter.

The cell suspension was passed through a 50 µm celltrix filter (Sysmex-Partec # 04-0042-2317) immediately before FACsorting the GFP+ NSCs using a MoFlo XDP cell sorter (Beckman Coulter) in the NYU Cytometry core facility. NSCs harvested from *Gli1*^*NULL*^ mice treated with corn oil served as negative controls for FACsorting. A forward scatter vs. side scatter dot plot was first used to gate the primary cell population and eliminate dead cells and debris. The gating was further refined to exclude doublets to finally select GFP+ cells (Fig. 2, Gating strategy exemplified by magenta arrows). In order to minimize any perturbation of gene expression, we performed cell sorting based on cell size and GFP expression immediately after obtaining a single cell suspension without manipulating the cells. To prevent the perturbation of gene expression, we did not add a viability dye to the cells during the FACsorting. However, we used 10 µl of the sorted GFP+ cells in phosphate buffered saline (PBS) for trypan blue cell viability assay and quantified the clear viable cells along with the blue stained dead cells using a hemocytometer^42^. All our FACsorted samples consisted of more than 95% viable cells. On average, the cell sorting yielded 17,666.7 ± 4163.3 GFP+ cells in the *Gli1*^*HET*^ healthy group on control diet, 7,833.3 ± 2,112.7 GFP+ cells in the *Gli1*^*HET*^ demyelination group on cuprizone diet, 41,333.3 ± 5033.2 GFP+ cells in the *Gli1*^*NULL*^ healthy group on control diet and 34,000 ± 10,816.7 GFP+ cells in the *Gli1*^*NULL*^ demyelination group on cuprizone diet.

### Total RNA isolation

The GFP positive neural stem cells were collected in RNA extraction buffer (Arcturus PicoPure RNA isolation kit, Applied Biosystems # 12204-01) after FACsorting and total RNA was isolated following the manufacturer’s instructions. The isolated RNA was eluted in 15 µl of elution buffer following on column DNase1 treatment using RNase-free DNase set (Qiagen # 79254). The total RNA was assessed for RNA integrity with a bioanalyzer (Agilent) in the NYU Genome Technology Center (Table 1). RNA samples with a RIN of more than 8 were submitted to NYU Genome Technology Center for Next Generation Sequencing (NGS).

**Table 1:** RNA quality of samples.

RNA quality		
Condition	RNA conc (pg/ul)	RNA Integrity Number (RIN)
Gli1-Het control diet-1	423	10
Gli1-Het control diet-2	931	8.5
Gli1-Het control diet-3	377	8.8
Gli1-Het cuprizone diet-1	186	8
Gli1-Het cuprizone diet-2	1209	9
Gli1-Het cuprizone diet-3	471	9
Gli1-Null control diet-1	1124	10
Gli1-Null control diet-2	1890	10
Gli1-Null control diet-3	927	8.6
Gli1-Null cuprizone diet-1	2041	10
Gli1-Null cuprizone diet-2	993	9.1
Gli1-Null cuprizone diet-3	1319	10

The RNA concentration and RNA integrity number (RIN) of all the samples.

### Library preparation and RNA sequencing

The mRNA was purified and cDNA was synthesized with the mRNA fragments as templates using SMART-Seq V4 RNA kit (Clontech # 634889). The sequencing libraries were generated using Nextera XT DNA Library Preparation Kit (Illumina #FC-131-1024), which uses an engineered transposome to simultaneously fragment and barcode the cDNA, adding unique short adapter sequences. The barcoded cDNAs were then amplified by 6 cycles of PCR and their quality was checked for size, quantity and integrity by TapeStation system (Agilent) as well as qPCR for precise quantification. The generated libraries were then mixed for multiplexing on 3 lanes and sequenced on Illumina HiSeq 2500 platform with 150 bp paired end modules, which yielded mapped reads in the range of 16–39 million per sample.

### Data transformation and downstream analysis

The data were transformed and analyzed by a bioinformatician in the NYU Genome Technology Center. Briefly, the raw sequencing data files were initially processed using FASTQC v.0.11.5 in order to perform quality control checks. Subsequently the Fastq files were aligned to the mm10 mouse reference genome with TopHat v2.1.1. About 93% to 96% of the RNAseq reads, including R1 reads in the 5′ to 3′ forward direction and R2 reads in the reverse direction, mapped to the mouse genome using the Ensembl gene annotation system^43^. The aligned bam files were then quantified for gene expression values utilizing HTSeq-count 0.11.1. Finally DESeq 2 v1.12 was used for analysis of the quantified count matrix^44^. Genes that had an adjusted p-value (False Discovery Rate) of less than 0.05 were considered significantly different for respective pairwise comparisons.

## Data Records

The sequencing data have been deposited in NCBI’s Gene Expression Omnibus and are accessible through GEO Series accession number GSE162683^45^. Table 2 lists the accession number for each sample. The data related to RNA quality, barcode lane statistics, RNA read counts, alignment statistics, DEseq 2 expression counts and the data comparison between the groups have been deposited in Figshare^46^.

**Table 2:** NCBI GEO accession numbers of samples.

NCBI Geo accession number	
Samples	Accession
Gli1-Het control diet-1	GSM4956878
Gli1-Het control diet-2	GSM4956879
Gli1-Het control diet-3	GSM4956880
Gli1-Het cuprizone diet-1	GSM4956881
Gli1-Het cuprizone diet-2	GSM4956882
Gli1-Het cuprizone diet-3	GSM4956883
Gli1-Null control diet-1	GSM4956884
Gli1-Null control diet-2	GSM4956885
Gli1-Null control diet-3	GSM4956886
Gli1-Null cuprizone diet-1	GSM4956887
Gli1-Null cuprizone diet-2	GSM4956888
Gli1-Null cuprizone diet-3	GSM4956889

Accession number of the samples included in the the NCBI GEO submission GSE162683.

## Technical Validation

### FACsorting

Since *Gli1*^*NULL*^ mice carry two copies of the *CreERT2* allele, they would be expected to have a higher probability of tamoxifen independent leaky GFP expression. Hence, we used cells isolated from the brains of *Gli1*^*NULL*^ mice but injected with corn oil instead of tamoxifen such that GFP is not expressed, as our negative control for FACsorting. These GFP negative cells were used to set up the gating parameters before GFP positive cells were sorted which ensured that only GFP positive neural stem cells were collected after sorting (Fig. 2).

### RNA quality

The quality of total RNA was assessed by RNA Integrity Number (RIN) using the Agilent Bioanalyzer. Following the standard practice in NGS, we used RNA with RIN >8 for our sequencing analysis^47^ (Table 1).

### Genotypic and phenotypic assessment

**Fig. 3:**
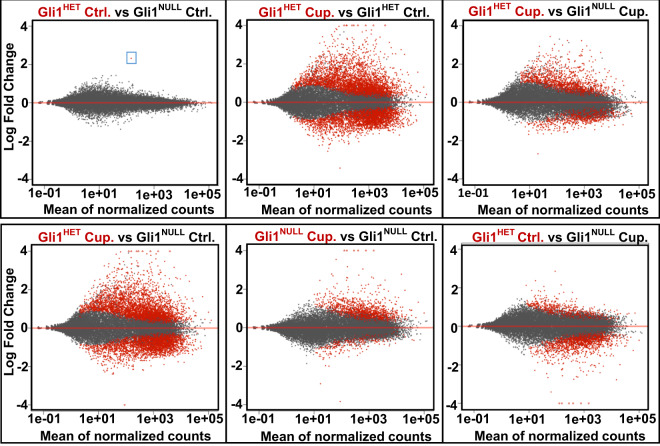
Visualization of differential gene expression in *Gli1*^*Het*^ and *Gli1*^*Null*^ NSCs. MA plots of the RNAseq data show the differences in expression of genes between the two genotypes on control and cuprizone diets. The blue box highlights the only gene differentially expressed between *Gli1*^*Het*^ and *Gli1*^*Null*^ NSCs in the control diet groups, corresponding to Gli1 itself. Ctrl.- Control diet group, Cup.-Cuprizone diet group.

Before harvesting the neural stem cells, we collected the tail tissue from all the mice following euthanasia. These tissues were used for PCR analysis to reconfirm the genotypes of the mice used to isolate neural stem cells. We also obtained a higher number of GFP positive cells from *Gli1*^*Null*^ mice compared to *Gli1*^*Het*^ mice, consistent with higher proliferation rates of NSCs in the *Gli1*^*Null*^ mice^27^, further validating their phenotype. An analysis of differential gene expression in healthy *Gli1*^*Het*^ NSCs vs. healthy *Gli1*^*Null*^ NSCs showed a significant difference in the expression of only one gene i.e. *Gli1*, which not only confirmed the genotype but also confirmed the well-established lack of phenotype in the healthy *Gli1*^*Null*^ mice^48^ (Fig. 3). In contrast, there were substantial differences (i.e. 1507 genes) in the transcriptomes of *Gli1*^*Het*^ vs. *Gli1*^*Null*^ NSCs fed with cuprizone diet for 3 weeks (Fig. 3, Table 3), consistent with their very distinct responses to demyelination. Many of these genes were downregulated in the nulls in agreement with the role of Gli1 as a transcriptional activator^49,50^. In the forebrain, Gli1 is expressed by neural stem cells in the SVZ and by mature astrocytes in the parenchyma outside the SVZ excluding the white matter tracts^26,51^. Consistently, the expression of stem cell marker genes (*Sox2, GFAP, Nestin*) were higher than genes expressed in differentiated cells (*APC, Htra1, S100β, PDGFRα, SYNGAP1*) in both *Gli1*^*HET*^ and *Gli1*^*NULL*^ healthy control NSCs.

**Table 3:** Number of differentially expressed genes.

Number of differentially expressed genes					
Comparison	No. of genes: P < 0.05	No. of upregulated genes: P < 0.05	No. of downregulated genes: P < 0.05	No. of genes: P < 0.05 & FC > 2	No. of genes: P < 0.05 & FC < 0.5
Gli1^HET^ Control vs Gli1^NULL^ Control	1	1	0	1	0
Gli1^HET^ Cuprizone vs Gli1^HET^ Control	6442	3481	2961	1447	525
Gli1^HET^ Cuprizone vs Gli1^NULL^ Cuprizone	1507	955	552	500	64
Gli1^HET^ Cuprizone vs Gli1^NULL^ Control	6447	3499	2948	1482	526
Gli1^NULL^ Cuprizone vs Gli1^NULL^ Control	1209	1015	194	294	34
Gli1^HET^ Control vs Gli1^NULL^ Cuprizone	1730	436	1294	86	242

Genes that had an adjusted p-value (False Discovery Rate) of less than 0.05 and fold change of more than 2 or less than 0.5 in the compared groups are quantified.

### Clustering of RNAseq data

**Fig. 4:**
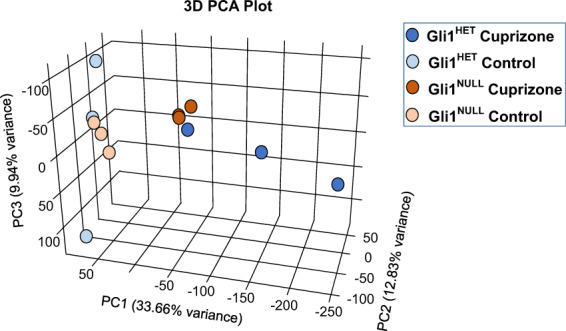
Principle component analysis (PCA) of the sequencing data. A 3D PCA plot shows segregation of the control diet samples from the cuprizone diet samples. The variance is higher in the Gli1^Het^ samples compared to the Gli1^Null^ samples.

**Fig. 5:**
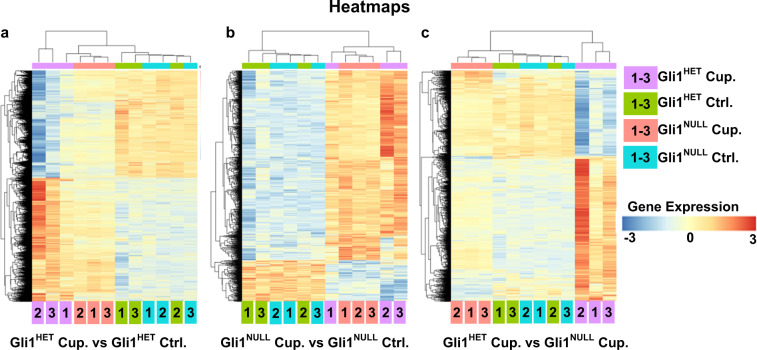
Heatmaps of the differentially expressed genes (FDR < 0.05). (**a**) Comparison of *Gli1*^*Het*^ NSCs following demyelination with cuprizone diet (Cup.) (purple) vs. healthy NSCs on control diet (Ctrl.) (green). (**b**) Comparison of *Gli1*^*Null*^ NSCs following demyelination with cuprizone diet (Cup.) (red) vs. healthy NSCs on control diet (Ctrl.) (blue). (**c**) Comparison of the *Gli1*^*Het*^ vs. *Gli1*^*Null*^ NSCs following demyelination with cuprizone diet (Cup). The sample numbers are mentioned inside the color coded box for each condition, at the bottom of the heatmap. The gene expression levels are color coded with the highest upregulated genes in red and the most down-regulated genes in blue.

A 3D Principle Component Analysis (PCA) plot visualized by Plotly (Fig. 4) showed clustering of the samples in the control diet groups from both *Gli1*^*Het*^ and *Gli1*^*Null*^ neural stem cells; similarly the cuprizone diet RNA samples from both genotypes clustered together but away from the control diet groups. We observed an increased variance in the *Gli1*^*Het*^ samples of both control and cuprizone diet groups. However, the heatmaps for the differentially expressed genes (FDR < 0.05) showed clustering of *Gli1*^*Het*^ and *Gli1*^*Null*^ samples in the control groups (Fig. 5). When we compared the differentially expressed genes (FDR < 0.05) between the cuprizone groups, the expression in the *Gli1*^*Null*^ samples appeared similar to the expression in the control groups (Fig. 5), suggesting that demyelination results in lesser perturbation of gene expression in *Gli1*^*Null*^ NSCs compared to *Gli1*^*Het*^ NSCs.

### Ingenuity pathway analysis

To examine the altered response of *Gli1*^*Null*^ NSCs to demyelination compared to *Gli1*^*Het*^ NSCs, we analyzed 1507 differentially expressed genes (p < 0.05), between *Gli1*^*Het*^ and *Gli1*^*Null*^ cuprizone groups with Ingenuity Pathway Analysis (Qiagen, Build version: 448560 M) (Table 4). While neurological diseases scored highest amongst the perturbed networks, the cellular functions related to survival and development were amongst the top 5 significant differences between the 2 groups. Consistently, TP53 and TGFβ1 were the most significant upstream regulators and these pathways were predicted to be more active in *Gli1*^*Het*^ NSCs upon demyelination.

**Table 4:** Summary of Ingenuity Pathway Analysis of differentially expressed genes in *Gli1*^*HET*^ cuprizone vs *Gli1*^*NULL*^ cuprizone groups.

IPA Analysis: Gli1^HET^ Cuprizone vs Gli1^NULL^ Cuprizone		
Networks		Score
1. Developmental Disorder, Hereditary Disorder, Neurological Disease		46
2. Cellular Assembly and Organization, Cellular Function and Maintenance, Cellular Development		43
3. Cell Cycle, Cellular Assembly and Organization, DNA Replication, Recombination, and Repair		38
4. Lipid Metabolism, Small Molecule Biochemistry, Auditory Disease		36
5. Organ Morphology, Organismal Development, Organismal Injury and Abnormalities		36
**Physiological System Development and Function**	**p Value**	**# Genes**
Organismal Survival	6.94E-07 - 5.57E-25	417
Tissue Development	3.23E-06 - 3.98E-21	551
Tissue Morphology	3.78E-06 - 2.74E-16	399
Embryonic Development	3.23E-06 - 6.79E-15	357
Organismal Development	3.78E-06 - 6.79E-15	551
**Molecular and Cellular Functions**	**p Value**	**# Genes**
Cellular Growth and Proliferation	3.13E-06 - 8.04E-26	645
Cell Morphology	3.74E-06 - 3.28E-25	458
Cell Death and Survival	3.51E-06 - 1.91E-21	515
Cellular Assembly and Organization	1.41E-06 - 1.04E-17	276
Cellular Function and Maintenance	3.74E-06 - 1.04E-17	453
**Upstream Regulators**	**p-value of overlap**	**Predicted Activation**
TP53	9.68E-25	Activated
TGFβ1	9.99E-19	Activated
TNF	1.38E-15	Activated
PDGF BB	9.79E-15	Activated
KRAS	1.88E-14	
**Top 10 Upregulated Genes (Log 2-fold expression value)**	**Top 10 Downregulated Genes (Log 2-fold expression value)**	
AHNAK2 (↑3.438)	GJD2 (↓−2.684)	
CLCF1 (↑3.226)	DENND1C (↓−1.647)	
ANXA3 (↑3.219)	MAG (↓−1.637)	
ADM (↑3.027)	ADGRL4 (↓−1.603)	
GPNMB (↑2.986)	BARD1 (↓−1.558)	
IGFBP3 (↑2.944)	LRRTM3 (↓−1.553)	
PLAUR (↑2.923)	GRM1 (↓−1.539)	
ARHGAP22 (↑2.816)	LOC102634852 (↓−1.501)	
FLNC (↑2.757)	PGBD5 (↓−1.494)	
SYTL2 (↑2.722)	Ly6a (↓−1.481)	

Top 5 perturbed networks, physiological system functions, molecular and cellular functions and upstream regulators are listed in addition to the top 10 upregulated and downregulated genes in *Gli1*^*HET*^ cuprizone NSCs.

## Data Availability

The codes used for aligning the RNAseq reads to the mm10 mouse reference genome with TopHat v2.1.1, quantifying the read counts using HTSeqCount, comparison of the gene expression using DESeq 2, and PCA analysis using Plotly 3-D PCA, are available in Figshare^46^.
